# A comparison of three pH control methods for revealing effects of undissociated butyric acid on specific butanol production rate in batch fermentation of *Clostridium acetobutylicum*

**DOI:** 10.1186/2191-0855-3-3

**Published:** 2013-01-07

**Authors:** Xuepeng Yang, Maobing Tu, Rui Xie, Sushil Adhikari, Zhaohui Tong

**Affiliations:** 1 Forest Products Lab and Center for Bioenergy and Bioproducts, Auburn University, 520 Devall Drive, Auburn, AL, 36849, USA; 2 Department of Biosystems Engineering, Auburn University, Auburn, AL, 36849, USA; 3 Department of Agriculture and Biological Engineering, University of Florida, Gainesville, FL, 32611, USA

**Keywords:** Butanol, Butyric acid, Fermentation, Undissociated butyric acid, *Clostridium acetobutylicum*

## Abstract

pH control has been essential for butanol production with *Clostridium acetobutylicum*. However, it is not very clear at what pH level the acid crash will occur, at what pH level butanol production will be dominant, and at what pH level butyric acid production will be prevailing. Furthermore, contradictory results have been reported about required acidic conditions for initiation of solventogenesis. In this study, with the aim of further understanding the role of undissociated butyric acid in butanol production, we investigated the correlation between undissociated butyric acid concentration and specific butanol production rate in batch fermentation of *Clostridium acetobutylicum* by comparing three pH control approaches: NaOH neutralization (at 12, 24 or 36 h), CaCO_3_ supplementation (2, 5, or 8 g/l) and NaOAc buffering (pH 4.6, 5.0 or 5.6). By neutralizing the fermentation pH to ~5.0 at different time, we observed that neutralization should take place at the beginning of exponential phase (12 h), and otherwise resulting in lower concentrations of undissociated butyric acid, cell biomass and final butanol. CaCO_3_ supplementation extended cell growth to 36 h and resulted in higher butyrate yield under 8 g/L of CaCO_3_. In the NaOAc buffering, the highest specific butanol rate (0.58 h^−1^) was associated with the highest undissociated butyric acid (1.92 g/L). The linear correlation of the undissociated butyric acid with the specific butanol production rates suggested the undissociated butyric acid could be the major driving force for butanol production.

## Introduction

Butanol is one of the promising advanced biofuels and an important intermediate in chemical synthesis. It is being pursued by industry and the U.S. government (the DOE, USDA and NSF) for the next generation of alternative fuels (Hess 2006; Li et al. 2011a; Li et al. 2011b; Milne et al. 2011; Qureshi and Ezeji 2008). Butanol, as one of the alternative biofuels, has several advantages over ethanol (the main transportation biofuel candidate) such as low vapor pressure and tolerance to water contamination (Hess 2006). Butanol production from sugars by fermentation is known as “acetone-butanol-ethanol fermentation or ABE fermentation” (Jones and Woods 1986). The typical products ratio from this process with *Clostridium acetobutylicum* is A: B: E = 3:6:1. *C. acetobutylicum* is capable of fermenting hexose and pentose sugars to butanol, but solvent yields and fermentation rates have varied depending on strains and fermentation conditions (Qureshi et al. 2008). The fermentation process by *C. acetobutylicum* is divided into two distinct phases: rapid cell growth and production of butyric acids take place in a first acidogenic phase accompanied by pH decrease, which is then replaced by a second solventogenic phase in which butanol and other solvents (mainly acetone and ethanol) are produced, leading to pH increase (Bryant and Blaschek 1988; Holt et al. 1984; Huang et al. 1986; Husemann and Papoutsakis 1988; Monot et al. 1984).

Previously, extensive research has been focused on the mechanisms of “acid crash” and initiation of solventogenesis (Martin et al. 1983; Wang et al. 2011; Zverlov et al. 2006). Acid crash occurs in pH uncontrolled fermentation and results in the cessation of glucose uptake, acids production and butanol production (Maddox et al. 2000). The overproduction of undissociated acids (>60 mM) with low pH could be the main cause for acid crash (Maddox et al. 2000). Recently, formic acid has also been suggested to trigger the acid crash of butanol production by *C. acetobutylicum* (Wang et al. 2011). Initiation of solventogenesis has been correlated well with the concentration of undissociated butyric acid (Huang et al. 1986; Husemann and Papoutsakis 1988; Monot et al. 1984). It has been suggested that solvent production initiated at the minimum of 1.5 g/L of undissociated butyric acid (Monot et al. 1984). A linear correlation has been found between butanol and undissociated butyric acid concentration at the onset of solvent formation (Husemann and Papoutsakis 1988). The addition of acetate, butyrate and propionate could improve the final solvent yields, but not much on the initiation of solventogenesis (Fond et al. 1985; Husemann and Papoutsakis 1990; Yu and Saddler 1983). However, the solvent formation could also be initiated at neutral pH (6.8–7.0) with the supplementation of high concentration of acetate plus butyrate (George and Chen 1983; Holt et al. 1984).

It is believed that the accumulation of a threshold amount of butyric acid initiates the solvent production. Particularly, the undissociated butyric acid excreted in the fermentation media re-enters the cells and serves as the precursor for butanol production (Bryant and Blaschek 1988; Husemann and Papoutsakis 1988). However, the excess of acids can be produced in the batch fermentation of *C. acetobutylicum* without pH control, and subsequently causes the cessation of glucose utilization (Maddox et al. 2000). Therefore, pH control has been essential for butanol production with *C. acetobutylicum* (Bryant and Blaschek 1988; Huang et al. 1986; Monot et al. 1984; Roos et al. 1985). However, it is not very clear at what pH level the acid crash will occur, at what pH level butanol production will be dominant, and at what pH level butyric acid production will be prevailing. Also, contradictory results have been reported that acidic conditions are not required for initiation of solventogenesis (George and Chen 1983). In this study, with the aim of improving butanol production yield, we investigated the effects of different pH control methods on butanol and butyric acid production in batch fermentation by *C. acetobutylicum.* In addition, we explored the potential mechanisms for the persistent butanol production under effective pH control.

## Material and methods

### Microbial strain and medium

*Clostridium acetobutylicum* (ATCC 824) was used in this study. The strain was maintained on Reinforced Clostridia Medium (RCM) with the addition of agar in a RT Anaero-Indicator. Isolated colony was cultured anaerobically in 50 mL of 38 g/L of RCM at 35°C for 24 h and left aerobically in ambient temperature overnight for the production of spores. The spores were washed and re-suspended in sterile water as the pre-inoculum (OD_600_ = 2). Before inoculation, the pre-inoculum was treated with heat shock at 80°C for 10 min. The heat shocked spores were cultured anaerobically into 50 mL of RCM liquid medium at 35°C for 12 h as seed inoculum. 5 mL of the seed inoculum was added into the 50 mL fermentation broth. All the culture experiments were conducted in 125 mL serum bottles. All the media were autoclaved at 121°C for 20 min before the inoculation. Cell density was measured by a UV–vis spectrometer at 600 nm. The dry cell weight (DCW) was calculated from the optical density based on the following equation DCW (g/L) = 0.3 A_600_.

### Fermentation and pH control methods

The fermentation was carried out in 125 mL serum bottles with 50 mL of P2 medium containing (g/L): glucose, 50; yeast extract, 1.0; ammonium acetate, 2.2; KH_2_PO_4_, 0.5; K_2_HPO_4_, 0.5; MgSO_4_ · 7H_2_O, 0.2; MnSO_4_ · 7H_2_O, 0.01; FeSO_4_ · 7H_2_O, 0.01; NaCl, 0.01. All the media were bubbled through nitrogen for 10 min to remove oxygen and autoclaved at 121°C for 20 min before fermentation inoculation. During the fermentation, samples were taken at regular intervals for analysis. Three different pH controlling methods were examined for their effects on fermentation by *C. acetobutylicum*. In the NaOH neutralization, 5 mL of 0.5 M NaOH was added into the fermentation broths at 12 h, 24 h, or 36 h respectively during the fermentation to adjust the pH to ~5.0. In the CaCO_3_ supplementation, 2 g/L, 5 g/L or 8 g/L of CaCO_3_ powder was added into the media to control the pH at the beginning of fermentation. In the sodium acetate buffering, the initial fermentation pH was controlled at 4.6, 5.0, or 5.6 by varying the ratio of HOAc/NaOAc. All the treatments were conducted in duplicate. Fermentation broth without pH control was used as a control.

### Analytical methods

Glucose concentration was determined by HPLC or using the DNS method. Butanol and butyric acid were analyzed by a Varian 3800 Gas Chromatography equipped with a Varian CP8400 autosampler, splitless injector system, and flame ionization detector. The separation was conducted using a Stabilwax-DA column fitted with a 5 m deactivated guard column. The separation conditions were used by a previously described method (Robinson et al. 2002). Butanol and butyric acid were quantified by chromatographic grade standards. pH was determined using pH test strips (BDH®, pH range 3.6–6.1, pH graduation 0.3/0.5).

## Results

### Effect of NaOH neutralization on cell growth, butanol and butyric acid production by *C. acetobutylicum*

**Figure 1:**
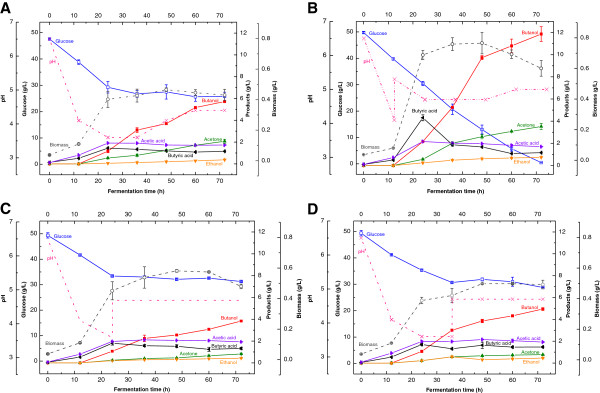
**Effect of NaOH neutralization on butanol production by *C. acetobutylicum *(A: fermentation control without pH control; B neutralization at 12 h; C: neutralization at 24 h; D: neutralization at 36 h).**

To determine at what time and pH level the acid crash will occur, we added 5 mL of 0.5 M NaOH at 12 h, 24 h, and 36 h individually during the fermentation to control the pH between 4.7 and 5.3. These three time points corresponded to the beginning of the exponential phase, the end of the exponential phase, and the stationary phase of cell growth. The results of butanol and butyric acid yields were given in Table 1 and further details of pH profiles, cell growth, glucose consumption and butanol production were shown in Figure 1. Without pH control (Figure 1A), the fermentation pH dropped to 4.1 at 12 h, 3.6 at 24 h, and remained at 3.6 until 36 h. After that, the fermentation pH started to increase and reached 4.4 at 72 h. The cells grew slowly in the first 12 h of lag phase, and then increased dramatically from 12 to 24 h (the exponential phase). When the pH dropped to approximately 3.6, the cell growth ended at 24 h and the cell biomass reached 0.40 g/L. Similar results have been reported previously when pH uncontrolled batch fermentation of *C. acetobutylicum* was performed in a fermentor (Husemann and Papoutsakis 1990). The butanol production started from 12 h and slowly increased to 5.7 g/L at 72 h. More than 50% of glucose was not consumed due to the potential acid crash. The acid crash seemed to take place before 24 h and the glucose consumption was ceased afterwards. This phenomenon can be described more precisely as “acid flush” because the excessive acids or low pH cause a weak fermentation in the solventogenic phase, rather “crash” solvent production and fermentation completely (Figure 1A). 

**Table 1:** **Effect of different pH regulation methods on cell growth and butanol production by *C. acetobutylicum***

**pH control**	**NaOH neutralization**			**CaCO**_**3**_**supplementation**			**NaOAc buffering (pH)**		
	12 h	24 h	36 h	2 g/L	5 g/L	8 g/L	4.6	5.0	5.6
pH at 24 h^a^	4.7 ± 0.0	3.6 ± 0.0	3.6 ± 0.0	4.4 ± 0.0	4.7 ± 0.0	5.3 ± 0.0	4.1 ± 0.0	4.4 ± 0.0	5.0 ± 0.0
C_butanol_^b^	11.9 ± 0.7	3.8 ± 0.0	4.9 ± 0.1	8.8 ± 0.4	10.8 ± 0.1	7.9 ± 0.4	6.5 ± 0.3	12.3 ± 0.9	8.3 ± 0.4
C_butyric acid_^c^	4.3 ± 0.3	1.8 ± 0.2	1.7 ± 0.1	4.0 ± 0.3	5.4 ± 0.5	6.2 ± 0.2	2 ± 0.3	2.7 ± 0.5	4.0 ± 0.2
Biomass	0.69 ± 0.03	0.45 ± 0.06	0.39 ± 0.02	0.45 ± 0.05	0.41 ± 0.02	0.53 ± 0.01	0.51 ± 0.03	0.63 ± 0.02	0.70 ± 0.04
C_UBA_^d^	2.18 ± 0.14	1.0 ± 0.08	1.6 ± 0.09	2.68 ± 0.18	3.01 ± 0.26	1.50 ± 0.05	1.70 ± 0.28	1.92 ± 0.32	1.32 ± 0.07
q_p_	0.41 ± 0.02	0.22 ± 0.09	0.42 ± 0.02	0.49 ± 0.06	0.52 ± 0.01	0.25 ± 0.02	0.44 ± 0.01	0.58 ± 0.01	0.36 ± 0.02

^a^ pH at 24 h: pH values at 24 h of fermentation. ^b^ C_butanol_ (g/L): Final butanol concentration at 72 h. Biomass (g/L): Cell biomass at 24 h. ^c^ C_butyric acid_: Butyric acid concentration at 24 h (peak concentration). C_UBA_ (g/L): Concentration of undissociated butyric acid at 24 h.

q_p_: Specific butanol production rates (between 24 and 36 h, h^−1^) were calculated based on the following equation: q_p_ = (C_butanol,36h_ − C_butanol,24h_)/(12 × X_cell biomass,24h_).

When we neutralized the pH to 5.3 with NaOH at 12 h, the glucose was completely consumed and the final butanol yield increased dramatically to 11.9 g/L (Figure 1B). The pH profile showed the fermentation pH was maintained between 4.7 and 5.0 and the cell biomass reached 0.68 g/L at 24 h, and increased by 70% as compared to the control. It appeared that acid flush was overcome under this scenario. However, when we neutralized the fermentation pH at 24 h and 36 h respectively, the glucose consumption was still ceased at 24 h and the butanol yields were very low at 72 h, both cases were similar to the fermentation control. It indicated the acid flush probably occurred early in the exponential phase (fermentation 12–24 h) in the pH range of 3.6–4.1, which likely is the reason why the later neutralization could not help improve glucose consumption and butanol production.

As for butyric acid production, with the NaOH neutralization at 12 h, the concentration of butyrate increased significantly from 1.4 g/L (without pH control) to 4.3 g/L at 24 h (Figures 1A and B), and then decreased significantly for butanol production. It appeared that more butyrate was converted into butanol when the fermentation pH is controlled around 4.7. Our results agreed well with the previous report that a threshold amount of undissociated butyric acid was required to start the solventogenesis (Husemann and Papoutsakis 1988; Monot et al. 1984). Failing to control the pH at 12 h was presumed to cause deactivation of key enzymes for acid and butanol production and even kill the cells (Welch et al. 1989). As a result, neutralizing pH at 24 and 36 h after acid flush could not lead to any improvement of butanol production. This assumption was in agreement with the glucose utilization, which was stopped after 24 h in these two cases as well as in the fermentation control (Figure 1).

### Effect of CaCO_3_ supplementation on cell growth, butanol and butyric acid production by *C. acetobutylicum*

**Figure 2:**
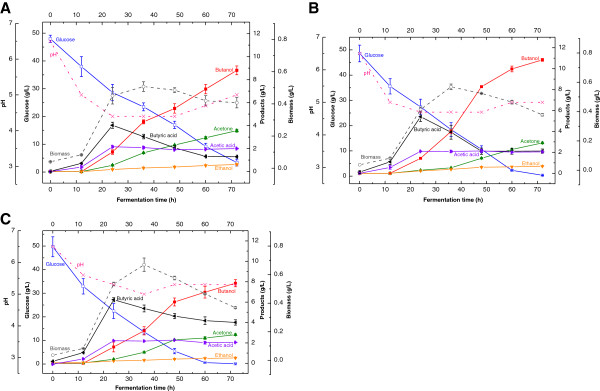
**Effect of CaCO**_**3 **_**supplementation on butanol production by *C. acetobutylicum *(A: CaCO**_**3 **_**supplementation at 2 g/L; B CaCO**_**3 **_**supplementation at 5 g/L; C: CaCO**_**3 **_**supplementation at 8 g/L).**

CaCO_3_ has been used to neutralize organic acids during fermentation and to maintain pH at a certain range (Huang et al. 2005; Vandak et al. 1997). To evaluate its effect on fermentation by *C. acetobutylicum*, we examined the effects of CaCO_3_ supplement (2, 5, 8 g/L) on butanol and butyric acid production by *C. acetobutylicum*. The initial pH was 6.5 for all the CaCO_3_ supplement treatments and the control. The results showed CaCO_3_ supplementation improved glucose uptake significantly resulting in nearly complete consumption of glucose (Figure 2A-C). Interestingly, we found that the CaCO_3_ supplementation at the concentration of 2, 5 or 8 g/L extended the cell growth to 36 h, and correspondingly increased the cell biomass from 0.43 g/L (control) to 0.51, 0.57 and 0.67 g/L at 36 h respectively. Consequently, we observed that the butanol production rates (between 36–48 h) increased dramatically from 0.05 g/L h (the control) to 0.10, 0.36 and 0.24 g/L · h when the CaCO_3_ supplementation of at 2, 5, and 8 g/L respectively. This indicated the strong correlation between cell biomass and butanol production, but high cell biomass could also result in higher butyrate, not butanol if the pH was not controlled within the appropriate range. For example, the higher CaCO_3_ supplementation (8 g/L) resulted in the highest cell biomass and butyrate concentration (4.0 g/L) at 72 h, while the butanol concentration was only 7.8 g/L. (Figure 2B and 2C). The highest butanol concentration reached 10.8 g/L at 72 h with the supplementation of 5 g/L CaCO_3_. At 24 h of fermentation, the pH was 4.4, 4.7, and 5.3 with the CaCO_3_ supplementation at 2, 5 and 8 g/L respectively. And the corresponding undissociated butyric acid was 2.7, 3.0 and 1.5 g/L at 24 h. The final butanol concentration (10.8 g/L) was higher when the fermentation pH (24 h) was kept at 4.7 than that (8.8 g/L) at pH 5.3. It suggested that butanol production was not only related to the cell biomass, but also correlated to the fermentation pH and the undissociated butyric acid. With the CaCO_3_ supplementation, the fermentation pH controlled between 4.4 and 5.3 at 24 h could overcome the acid flush, but higher undissociated butyric acid probably was a prerequisite for fast butanol production in the solventogenic phase. The results also indicated that keeping higher pH (5.3) in fermentation favored higher butyric acid production. Although the butyric acid could be converted into butanol in the later solventogenic phase; the final butyric acid concentration was still high. The similar results of pH effect on butyric acid production were reported in the previous research in which Monot et al. found that the medium with higher pH level produced more acids than the medium with lower pH level (Monot et al. 1984). 

### Effect of sodium acetate buffering on butanol and butyric acid production by *C. acetobutylicum*

**Figure 3:**
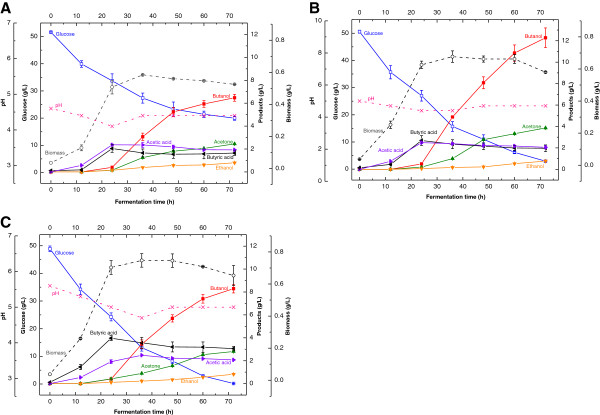
**Effect of NaOAc buffering on butanol production by *C. acetobutylicum *(A: NaOAc buffering at initial pH 4.6; B NaOAc buffering at initial pH 5.0; C: NaOAc buffering at initial pH 5.6).**

Buffering is another common method used in various fermentation processes to regulate pH (Bryant and Blaschek 1988). Therefore, the effects of different initial NaOAc buffering pH on butanol and butyric acid production by *C. acetobutylicum* were investigated to evaluate its effectiveness in comparison with those of CaCO_3_ supplementation and NaOH neutralization. Sodium acetate was chosen to buffer pH at 4.6, 5.0, and 5.6 initially by changing the ratio of NaOAc and HOAc. The results showed that NaOAc buffering at different initial pH could affect the fermentation pH profiles, cell growth, glucose consumption and butanol production significantly (Figure 3A-C). Buffering at initial pH 5.0 and 5.6 appeared to overcome the acid flush and enable complete glucose consumption, while buffering at initial pH 4.6 resulted in incomplete consumption of glucose and ~20 g/L glucose was not utilized at 72 h. At the end of exponential phase (24 h), the cell biomass reached 0.51, 0.63, 0.70 g/L and the fermentation pH dropped to 4.1, 4.4, and 5.0 respectively (for initial buffering pH at 4.6, 5.0 and 5.6). This indicated that pH 4.4 probably was a threshold for acid flush, pH below 4.4 resulted in the termination of glucose consumption. We also observed the lag phase was reduced significantly at the high initial buffering pH (5.0 and 5.6) compared with that at a low buffering pH (4.6). Buffering the initial pH at 5.0 produced the highest butanol concentration 12.3 g/L at 72 h. Although buffering the initial pH at 5.6 had the highest butyric acid concentration 4.0 g/L at 24 h, further conversion of butyric acid to butanol was slow and the butanol yield remained at a low level at the end of fermentation. Comparing three pH control methods revealed high similarities of the fermentation patterns between the sodium acetate buffering and the CaCO_3_ supplementation. In both methods, maintaining the pH above 5.0 at 24 h favored the butyric acid production, maintaining the pH between 4.4 and 4.7 at 24 h promoted the butanol production. More interestingly, this optimal pH favoring the butanol production was also reflected in the NaOH neutralization, in which the pH at 24 h was 4.7 by adjusting pH with NaOH at 12 h. The observed cell growth took place mainly between 12 and 24 h in fermentation, the cell biomass (DCW) increased from 0.1 g/L (at 12 h) to 0.7 g/L (at 24 h), and then remained almost constant. It suggested the first 12–24 h probably was the exponential phase of cell growth and butyric acid was produced associated with cell growth mainly in this phase. At the end of exponential phase, bacteria started to generate the butanol dehydrogenase, which was responsible for producing butanol. Therefore, controlling the fermentation pH between 4.4–4.7 at 12–24 h is essential for the maximum butanol production**.**

## Discussion

### Effects of pH control on the maximum biomass

**Figure 4:**
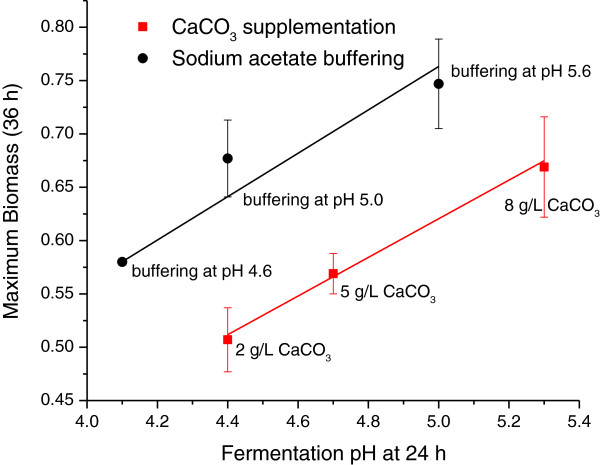
**Correlation between fermentation pH (24 h) and maximum biomass (36 h) in the batch fermentation of *C. acetobutylicum*.**

To seek the potential mechanisms involved in persistent butanol production, we observed both the cell biomass and the undissociated butyric acids correlated well with butanol production rates. The cell biomass could be related to the total enzyme activity in the later solvent production phase. Under the pH control with CaCO_3_ supplementation and NaOAc buffering, the cell biomass reached the maximum at 36 h. We observed a linear relationship between the maximum cell biomass and the fermentation pH at 24 h (Figure 4). This indicated that the higher pH at the end of exponential phases (in the pH range of 4.1–5.3), the higher the maximum biomass was achieved. This finding was consistent with the previous report with a pH-controlled batch fermentation of *C. acetobutylicum* using a fermentor (Monot et al. 1984), in which the increase of the fermentation pH between pH 4.5 and 5.5 enhanced the maximum biomass, but lowered the maximum biomass at a pH level higher than pH 6.0. In our work, we did not carry out the experiment at pH ≥ 6.0 because the low butanol yield would be produced at a higher pH. Figure 4 also showed that buffering the initial pH at 4.6 resulted in the fermentation pH 4.1 at 24 h and the lowest maximum biomass at 36 h. It indicated the low pH (~4.1) at the end of exponential phase inhibited the cell growth and the glucose utilization. The decease of pH from 4.6 to 4.1 was likely caused by the production of butyric acid and acetic acid in the acidogenic phase, where both acids reached their maximum concentrations at 24 h (Figures 1, 2, 3). Butyric acid and acetic acid have been reported as two major toxic products inhibiting cell growth of *C. acetobutylicum* based on their low inhibition constants (0.07 M and 0.19 M respectively)(Costa and Moreira 1983). As a result*,* microorganism probably develops a detoxification process involved a series of enzymes to convert butyric acid to butanol, and acetic acid to acetone in a solventogenic phase (Monot et al. 1984). Therefore, relatively low pH (<5.0) at the end of exponential phase is essential to maintain the persistent solvent production. More precisely, the concentration of undissociated butyric acid probably controls the butanol production rates in solventogenesis. 

### Effects of the undissociated butyric acid on specific butanol production rates

**Figure 5:**
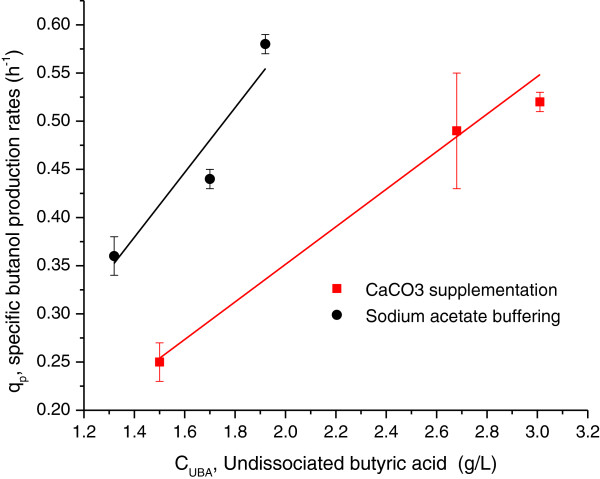
**Correlation between undissociated butyric acid concentration (24 h) and specific butanol production rate (between 24 and 36 h) in the batch fermentation of *C. acetobutylicum*.**

The correlation of the undissociated butyric acid (at 24 h) with the specific butanol production rates between 24 and 36 h was established in the fermentations with CaCO_3_ supplementation and NaOAc buffering. The butanol production rates increased dramatically between 24 and 36 h. The undissociated butyric acid reached the maximum concentration at 24 h (the end of exponential phase) except for the cases by adding 8 g/L of CaCO_3_ or buffering the initial pH at 5.6. In these two special cases, the maximum concentration of undissociated butyric acid was obtained at 36 h; consequently the butanol production rates were maximized between 36 and 48 h probably due to the extended cell growth. Figure 5 showed the specific butanol production rates (between 24 and 36) were a function of the undissociated butyric acid concentration (at 24 h). The linear relationship between them indicated the undissociated butyric acid was the major driving force for the production of butanol. Previously, others have reported a minimum amount (0.5 g/L–1.5 g/L) of undissociated butyric acid was required to initiate the solventogenesis (Husemann and Papoutsakis 1988; Monot et al. 1984). In our experiments, the onset of solvent production took place at much lower level of the undissociated butyric acid (0.20–0.30 g/L), which may attributed to the use of different media and pH controlled methods. The persistent butanol production could be more important than initial butanol production, since the persistent solvent production would be essential for a higher final butanol concentration at the end of fermentation. The specific butanol production rates at the end of exponential phase could represent the capability of persistent butanol production. For example, in the both cases of CaCO_3_ supplementation (5 g/L) and NaOAc buffering (pH 5.0), the higher the specific butanol production rates (0.52 h^−1^ and 0.58 h^−1^), the higher the final butanol concentration (10.8 g/L and 12.3 g/L) (Table 1). The cell biomass is essential for the evaluation of the persistent butanol productivity and therefore has been taken into accounts for the specific butanol production rates, because the enzymes (butanol dehydrogenase and CoA transferase) induced or produced for solventogenesis are related to the cell biomass. The undissociated acetic acid was suggested not required for solventogenesis (Husemann and Papoutsakis 1988). But the addition of acetate and propionate could enhance final solvent concentrations (Husemann and Papoutsakis 1990; Mattaelammouri et al. 1987). And the addition of butyrate demonstrated the potential to initiate the solvent production at pH 5 (Holt et al. 1984). The addition of butyrate probable increases the concentration of undissociated butyric acid, which can induce the corresponding enzymes synthesis and drive the butanol production (Husemann and Papoutsakis 1989). 

In this study, the pH control was essential for the improvement of butanol production by *C. acetobutylicum*, because the appropriate pH in the exponential phase could enhance the specific butanol production rates by producing higher undissociated butyric acid. NaOH neutralization was a simple method to control the pH, and the key time to adjust the fermentation pH is the beginning of exponential phase. CaCO_3_ supplementation resulted in relatively low content of cell biomass and high yield of butyrate. NaOAc buffering at pH 5.0 enabled a highest butanol production rate in the stationary phase. The linear correlation of the undissociated butyric acid with the specific butanol production rates suggests the undissociated butyric acid could be the major driving force for butanol production. The key to achieve high butanol concentration is to maintain a pH level around 4.7 and a higher level of undissociated butyric acid during the exponential phase of cell growth.

## Competing interests

The authors declare that they have no competing interests.

## References

[B1] Bryant DL Blaschek HP Buffering as a means for increasing growth and butanol production by clostridium-acetobutylicum J Ind Microbiol 1988 3 1 49 55 10.1007/BF01569442

[B2] Costa JM Moreira AR Growth-inhibition kinetics for the acetone-butanol fermentation Acs Sym Ser 1983 207 501 512

[B3] Fond O Mattaammouri G Petitdemange H Engasser JM The role of acids on the production of acetone and butanol by clostridium-acetobutylicum Appl Microbiol Biot 1985 22 3 195 200

[B4] George HA Chen JS Acidic conditions Are Not obligatory for onset of butanol formation by clostridium-beijerinckii (synonym, clostridium-butylicum) Appl Environ Microb 1983 46 2 321 327 10.1128/aem.46.2.321-327.1983 PMC239380 16346358

[B5] Hess G BP and dupont plan ‘biobutanol’ Chem Eng News 2006 84 26 9

[B6] Holt RA Stephens GM Morris JG Production of solvents by clostridium-acetobutylicum cultures maintained at neutral pH Appl Environ Microb 1984 48 6 1166 1170 10.1128/aem.48.6.1166-1170.1984 PMC241703 16346678

[B7] Huang L Forsberg CW Gibbins LN Influence of external pH and fermentation products on clostridium-acetobutylicum intracellular pH and cellular-distribution of fermentation products Appl Environ Microb 1986 51 6 1230 1234 10.1128/aem.51.6.1230-1234.1986 PMC239050 16347081

[B8] Huang LP Jin B Lant P Zhou JT Simultaneous saccharification and fermentation of potato starch wastewater to lactic acid by rhizopus oryzae and rhizopus arrhizus Biochem Eng J 2005 23 3 265 276 10.1016/j.bej.2005.01.009 15947951

[B9] Husemann MHW Papoutsakis ET Solventogenesis in clostridium-acetobutylicum fermentations related to carboxylic-acid and proton concentrations Biotechnol Bioeng 1988 32 7 843 852 10.1002/bit.260320702 18587795

[B10] Husemann MHW Papoutsakis ET Enzymes limiting butanol and acetone formation in continuous and batch cultures of clostridium-acetobutylicum Appl Microbiol Biot 1989 31 5–6 435 444

[B11] Husemann MHW Papoutsakis ET Effects of propionate and acetate additions on solvent production in batch cultures of clostridium-acetobutylicum Appl Environ Microb 1990 56 5 1497 1500 10.1128/aem.56.5.1497-1500.1990 PMC184438 2339898

[B12] Jones DT Woods DR Acetone-butanol fermentation revisited Microbiol Rev 1986 50 4 484 524 3540574 10.1128/mr.50.4.484-524.1986 PMC373084

[B13] Li SY Srivastava R Parnas RS Study of in situ 1-butanol pervaporation from a-B-E fermentation using a PDMS composite membrane: validity of solution-diffusion model for pervaporative a-B-E fermentation Biotechnol Progr 2011 27 1 111 120 10.1002/btpr.535 21312361

[B14] Li SY Srivastava R Suib SL Li Y Parnas RS Performance of batch, fed-batch, and continuous a-B-E fermentation with pH-control Bioresource Technol 2011 102 5 4241 4250 10.1016/j.biortech.2010.12.078 21227684

[B15] Maddox IS Steiner E Hirsch S Wessner S Gutierrez NA Gapes JR Schuster KC The cause of “acid crash” and “acidogenic fermentations” duping the batch acetone-butanol-ethanol (ABE-) fermentation process J Mol Microb Biotech 2000 2 1 95 100 10937493

[B16] Martin JR Petitdemange H Ballongue J Gay R Effects of acetic and butyric acids on solvents production by clostridium-acetobutylicum Biotechnol Lett 1983 5 2 89 94 10.1007/BF00132165

[B17] Mattaelammouri G Janatiidrissi R Junelles AM Petitdemange H Gay R Effects of butyric and acetic-acids on acetone butanol formation by clostridium-acetobutylicum Biochimie 1987 69 2 109 115 10.1016/0300-9084(87)90242-2 3032286

[B18] Milne CB Eddy JA Raju R Ardekani S Kim PJ Senger RS Jin YS Blaschek HP Price ND Metabolic network reconstruction and genome-scale model of butanol-producing strain clostridium beijerinckii NCIMB 8052 BMC Syst Biol 2011 5 130 10.1186/1752-0509-5-130 PMC3212993 21846360

[B19] Monot F Engasser JM Petitdemange H Influence of pH and undissociated butyric-acid on the production of acetone and butanol in batch cultures of clostridium-acetobutylicum Appl Microbiol Biot 1984 19 6 422 426 10.1007/BF00454381

[B20] Qureshi N Ezeji TC Butanol, ‘a superior biofuel’ production from agricultural residues (renewable biomass): recent progress in technology Biofuel Bioprod Bior 2008 2 4 319 330 10.1002/bbb.85

[B21] Qureshi N Saha BC Cotta MA Butanol production from wheat straw by simultaneous saccharification and fermentation using clostridium beijerinckii: part II - Fed-batch fermentation Biomass Bioenerg 2008 32 2 176 183 10.1016/j.biombioe.2007.07.005

[B22] Robinson JR Keating JK Boussaid AB Mansfield SM Saddler JS The influence of bark on the fermentation of douglas-fir whitewood pre-hydrolysates Appl Microbiol Biotechnol 2002 59 4 443 448 10.1007/s00253-002-1055-z 12172607

[B23] Roos JW Mclaughlin JK Papoutsakis ET The effect of pH on nitrogen supply, cell-lysis, and solvent production in fermentations of clostridium-acetobutylicum Biotechnol Bioeng 1985 27 5 681 694 10.1002/bit.260270518 18553724

[B24] Vandak D Zigova J Sturdik E Schlosser S Evaluation of solvent and pH for extractive fermentation of butyric acid Process Biochem 1997 32 3 245 251 10.1016/S0032-9592(96)00084-2

[B25] Wang SH Zhang YP Dong HJ Mao SM Zhu Y Wang RJ Luan GD Li Y Formic acid triggers the “acid crash” of acetone-butanol-ethanol fermentation by clostridium acetobutylicum Appl Environ Microb 2011 77 5 1674 1680 10.1128/AEM.01835-10 PMC3067271 21216898

[B26] Welch RW Rudolph FB Papoutsakis ET Purification and characterization of the nadh-dependent butanol dehydrogenase from clostridium-acetobutylicum (atcc 824) Arch Biochem Biophys 1989 273 2 309 318 10.1016/0003-9861(89)90489-X 2673038

[B27] Yu EKC Saddler JN Enhanced acetone-butanol fermentation by clostridium-acetobutylicum grown on D-xylose in the presence of acetic or butyric-acid FEMS Microbiol Lett 1983 18 1–2 103 107

[B28] Zverlov VV Berezina O Velikodvorskaya GA Schwarz WH Bacterial acetone and butanol production by industrial fermentation in the soviet union: use of hydrolyzed agricultural waste for biorefinery Appl Microbiol Biot 2006 71 5 587 597 10.1007/s00253-006-0445-z 16685494

